# Pseudotumor Cerebri Syndrome Without Headache in an Obese Male With Eight Restricted Cerebrospinal Fluid (CSF) Oligoclonal Bands: A Case Report

**DOI:** 10.7759/cureus.22024

**Published:** 2022-02-08

**Authors:** Muhammad Ismail Khalid Yousaf, Ping Shi, Rolando M Cordoves Feria, Mohammad Ravi Ghani, David A Robertson

**Affiliations:** 1 Neurology, University of Louisville School of Medicine, Louisville, USA

**Keywords:** headache disorders, idiopathic intracranial hypertension (iih), pseudotumor cerebri syndrome (ptcs), oligoclonal bands, all neurology

## Abstract

Pseudotumor cerebri syndrome (PTCS) is a condition caused by an abnormal elevation of intracranial pressure (ICH), which may be primary (idiopathic intracranial hypertension) or because of an identifiable secondary cause. We present a rare case of an obese male who complained of gradual bilateral vision loss for one year without headaches and tinnitus. On fundoscopy, he had high-grade bilateral papilledema and, on lumbar puncture, he had an elevated intracranial pressure of 260 mmH2O. Cerebrospinal fluid (CSF) was unique for eight restricted oligoclonal bands while extensive other demyelinating workup was negative. He was started on acetazolamide initially and subsequently proceeded with bilateral optic nerve sheath fenestration (ONSF) with mild improvement in the right eye and no improvement in the left eye. Although the causative mechanism of PTCS is a matter of debate, immune-mediated processes are one of the proposed mechanisms that may play a role in the pathophysiology of PTCS, evidenced by the presence of oligoclonal bands (OCBs) and pro-inflammatory markers in CSF. PTCS diagnosed in men and patients with OCBs poses an increased risk of vision loss as this case and literature documented. Therefore, prompt treatment through therapeutic lumbar punctures, acetazolamide therapy concurrently with weight loss, and surgical intervention in severe or refractory cases are necessary.

## Introduction

Pseudotumor cerebri syndrome (PTCS) is more common in obese females of childbearing age [1-2]; however, it can also occur in men with severe visual outcomes [1]. The visual loss could be gradual, in one or both eyes. Headaches are the most common symptoms of PTCS [2]. The pathophysiology of PTCS is a matter of ongoing debate. Possible favored mechanisms involve CSF flow system abnormalities, immune-mediated pathogenesis due to the presence of OCBs and other inflammatory markers in CSF, and the body's inflammatory response due to obesity. Revised diagnostic criteria for PTCS in adults and children is helpful to establish the PTCS diagnosis [3]. PTCS, although uncommon in men, can manifest without classic headaches and tinnitus, and an early ocular fundi exam is crucial, as chronic papilledema can lead to permanent visual loss.

## Case presentation

A 30-year-old, morbidly obese male (body mass index (BMI) of 59.26 kg/m²) with no significant past medical history presented to our multiple sclerosis (MS) clinic for suspicion of a demyelinating cause of PTCS after his primary neurologist and ophthalmologist found bilateral papilledema, with an opening lumbar puncture pressure of 260 mmH20 and eight restricted OCBs in CSF. His primary complaint was bilateral visual disturbances, which progressively worsened in the last year. His vision obscurations were not associated with headache, nausea, vomiting, dizziness, photophobia, and tinnitus. He also denied weakness, paresthesias, and gait problems. Although the patient had no recent history of known infections, hospital admissions, and tick bites, he felt extremely ill, tired, and had flu-like symptoms before visual symptoms appeared. At the time, he tested negative for coronavirus disease 2019 (COVID-19) disease. He was not taking any medications and denied taking any antibiotics before visual problems occurred.

On initial evaluation, he was hemodynamically stable and afebrile. His blood pressure (BP) was 166/94 mmHg and his pulse was 102 beats per minute (bpm). Complete blood count (CBC) and comprehensive metabolic panel (CMP) were within normal limits. C-reactive protein (CRP) 29.0 mg/L (< 10 mg/L) and erythrocyte sedimentation rate (ESR) 31 mm/hr (0-15 mm/hr) were elevated. Antinuclear cytoplasmic antibodies (ANCA) screen and antinuclear antibodies (ANA) screen were negative (Table 1).

**Table 1:** Serum laboratory tests and results NMO: Neuromyelitis optica; MOG: Myelin oligodendrocyte glycoprotein; CRP: C-reactive protein; ESR: Erythrocyte sedimentation rate; ANCA: Antineutrophil cytoplasmic antibodies; ANA (IFA): Antinuclear antibodies (immunofluorescence assay); SPEP: Serum protein electrophoresis

Laboratory Test	Results	Reference range
Complete blood count	Normal	Normal
Basic metabolic panel	Normal	Normal
Oligoclonal bands (common to CSF and Serum)	2 paired bands	Negative
Anti-MOG IgG	Negative	Negative
NMO/AQP4 autoantibodies IgG	Negative	Negative
CRP	29 ↑	<=10.0 mg/Lite
ESR	31 ↑	0 - 15 mm/Hr
ANCA Screen	Negative	Negative
ANA-IFA Screen	Negative	Negative
SPEP	Normal	Normal

He was fully alert and oriented on neurological examination and had fluent speech and intact comprehensive abilities. There were no signs of meningeal irritation. Cranial nerve (CN) testing revealed 3-5 mm pupils equal in size and reactive to light and accommodation, intact extraocular movements with no nystagmus, saccadic movement, or skew. The facial sensation was similar on both sides, with a strong jaw opening and a midline tongue. In addition, the shoulder shrug was symmetrical and hearing was intact. The rest of his neurological examination, including motor function, sensation, reflexes, coordination, and gait analysis, was within normal limits.

Fundus examination of the right eye revealed blurring of the superior margins of the optic disc corresponding with grade 2 papilledema, and the left eye exhibited paleness of optic disc correlating with grade 3 papilledema. Visual acuity was limited to finger counting in both eyes. Humphrey's vision test was significant for near to complete vision loss in the left eye.

Magnetic resonance imaging (MRI) of the brain and orbit didn't evidence any demyelinating disease; however, bilateral optic sheaths expansion, low lying cerebellar tonsils, empty sella, and stenosed transverse and sigmoid sinus on magnetic resonance venogram (MRV) correlated with elevated intracranial pressure. MRI cervical spine and thoracic spine were negative for structural abnormalities and demyelinating lesions. No abnormal enhancement was seen in either of the scans as mentioned above.

Lumbar puncture (LP) was done once and had an elevated opening pressure of 260 mmH20 (< 200 mmH20). CSF labs were relevant for eight CSF restricted oligoclonal bands, myelin basic protein elevation at 4.4 ng/mL, elevated immunoglobulin G (IgG) synthesis rate of 53.7 mg/day, high IgG index of 2.9, four nucleated cells, four red blood cells (RBCs), protein 41 mg/dL, glucose 79 mg/dL, and negative neuromyelitis optica (NMO) antibodies. In addition, in serum, anti-myelin oligodendrocyte glycoprotein (MOG) and neuromyelitis optica antibodies were negative as well (Table 2).

**Table 2:** CSF laboratory tests and results RBC: Red blood cell; CSF: Cerebrospinal fluid; NMO: Neuromyelitis optica; IgG: Immunoglobulin G

Laboratory Test	Result	Reference Values
Opening pressure	26 ↑	100-200 mm H_2_O
Oligoclonal bands ( CSF only)	8 ↑	Negative
Myelin basic protein	4.4 ↑	0.0 - 3.8 ng/mL
IgG synthesis rate	53.7 ↑	-9.9 TO +3.3 mg/day
IgG index	2.9 ↑	0.0 - 0.7
Alpha 1 Globulin	0.35 ↑	0.11 - 0.34 g/dL
Nucleated cells	4	0 - 5 /mm3
RBC	4	Negative
Protein	41	15.0 - 45.0 mg/dL
Glucose	79 ↑	40 - 70 mg/dL
CSF/Serum Albumin Index	4	< 9 is correlated with intact blood-brain barrier

The patient was initially treated with acetazolamide 500 mg/twice a day, started by his primary neurologist, and titrated weekly up to 1500 mg/twice a day. Unfortunately, his visual symptoms did not subside, and on our recommendation, he first underwent right eye optic nerve sheath fenestration (ONSF), and, after one month, left eye ONSF. As a result, his peripheral vision improved in the right eye, but no improvement in the left eye so far has been reported.

## Discussion

Pseudotumor cerebri syndrome (PTCS) or idiopathic intracranial hypertension (IIH) in men is rare, with a prevalence of 9% [1]. PTCS in men poses a twofold risk of developing visual loss compared to females [1]. As per the idiopathic intracranial hypertension treatment trial [2], secondary causes for increased intracranial pressure need to be ruled out comprehensively when considering PTCS in men and patients without headache and pulse synchronous tinnitus. There is a consensus regarding CSF opening pressure of ≥250 mmH2O in adults and ≥280 mmH2O in children (obese and sedated) to diagnose PTCS considering other clinical symptoms, including headache associated with PTCS [3-4]. We used Friedland's revised standards as published in Neurology, Journal of the American Academy of Neurology, while identifying and discussing PTCS in this case (Table 3) [3].

**Table 3:** A diagnosis of pseudotumor cerebri syndrome is definite if the patient fulfills criteria A–E. The diagnosis is considered probable if criteria A–D are met but the measured CSF pressure is lower than specified for a definite diagnosis PTCS: Pseudotumor cerebri syndrome; ICP: Intracranial pressure; MRI: Magnetic resonance imaging; CT: Computerized tomography; CSF: Cerebrospinal fluid

Diagnostic criteria for PTCS
A. Papilledema
B. Normal neurologic examination except for cranial nerve abnormalities
C. Neuroimaging: Normal brain parenchyma without evidence of hydrocephalus, mass, or structural lesion and no abnormal meningeal enhancement on MRI, with and without gadolinium, for typical patients (obese women), and MRI, with and without contrast, and MRV for others; if MRI is unavailable or contraindicated, contrast-enhanced CT may be used
D. Normal CSF composition
E. Elevated lumbar puncture CSF opening pressure (≥250 mmH20 in adults and ≥280 mmH20 in children [250 mmH20 if the child is not sedated and not obese]) in a properly performed lumbar puncture

Diagnosis of pseudotumor cerebri syndrome without papilledema
In the absence of papilledema, a diagnosis of pseudotumor cerebri syndrome can be made if B–E from above are satisfied, and in addition, the patient has a unilateral or bilateral abducens nerve palsy In the absence of papilledema or sixth nerve palsy, a diagnosis of pseudotumor cerebri syndrome can be suggested but not made if B–E from above are satisfied, and in addition at least 3 of the following neuroimaging criteria are satisfied:
1) Empty sella, 2) Flattening of the posterior aspect of the globe, 3) Distention of the perioptic subarachnoid space with or without a tortuous optic nerve, 4)Transverse venous sinus stenosis

**Figure 1:**
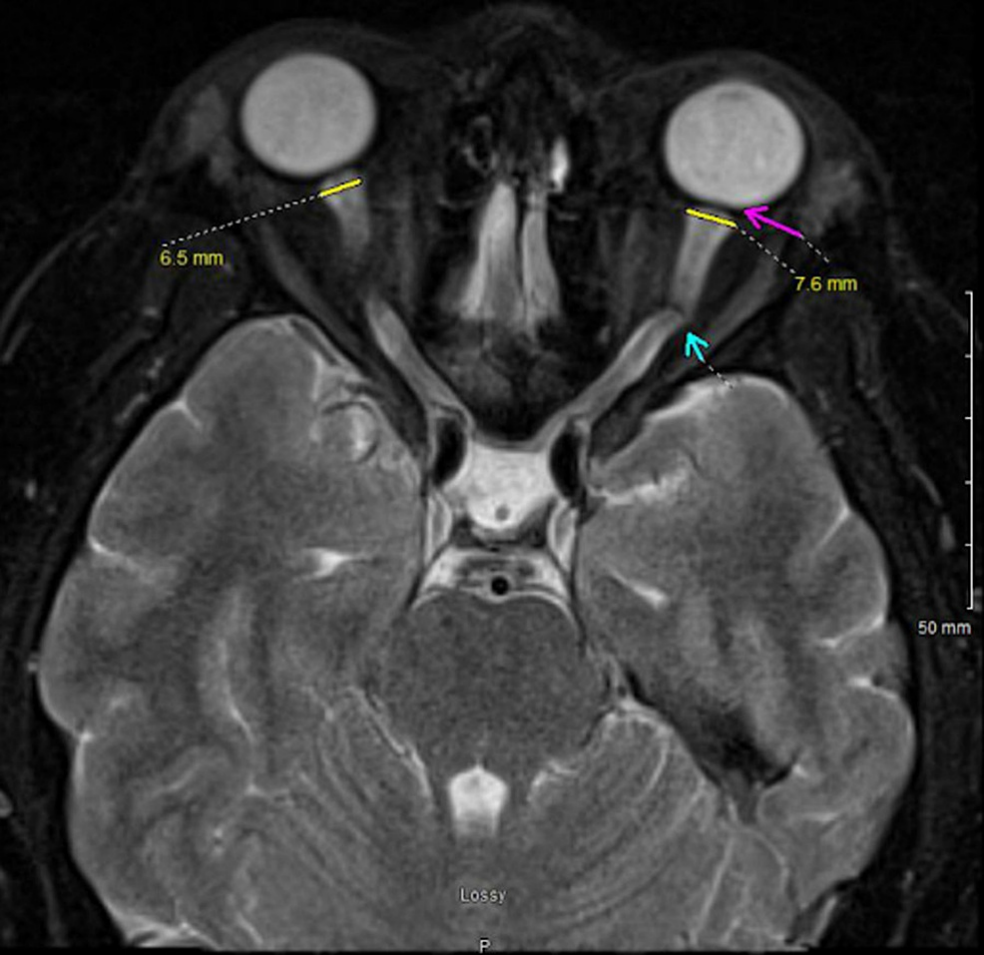
T2-weighted magnetic resonance (MR) image (axial view) showing bilateral expanded optic sheaths (normal range 5.17±1.34 mm to 3.55±0.82 mm) with optic nerve tortuosity (cyan arrow) and posterior globe flattening (magenta arrow), more prominent in the left eye

**Figure 2:**
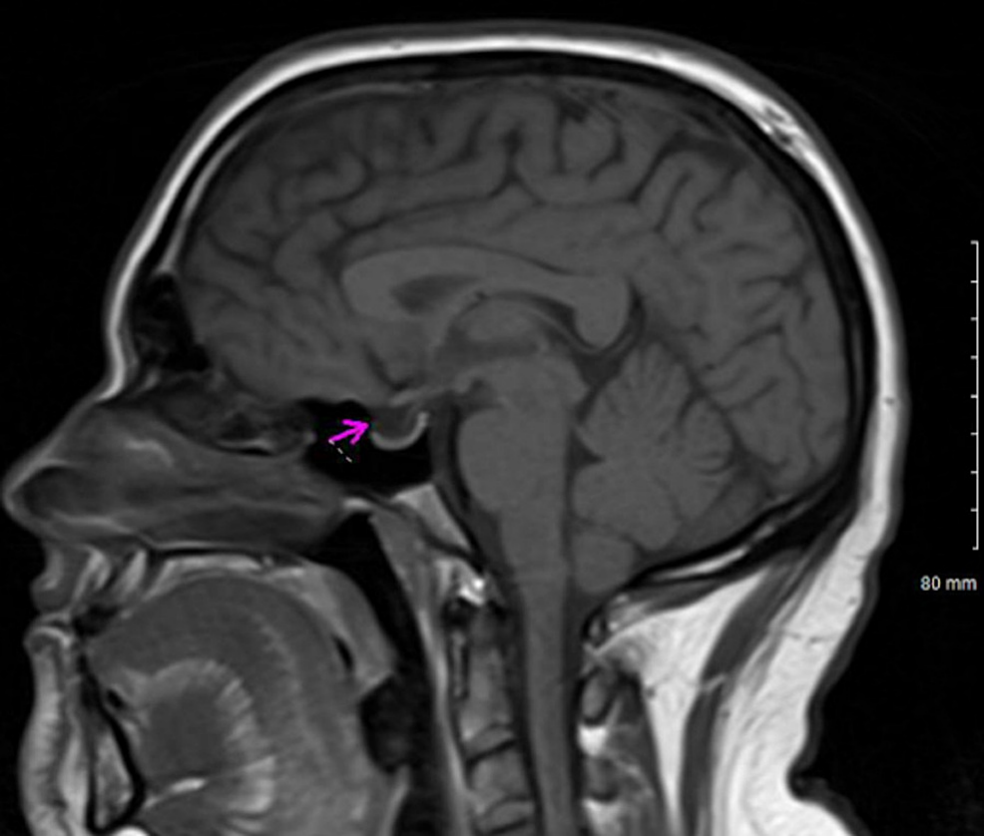
T1 (sagittal view) showing an empty sella (magenta arrow)

**Figure 3:**
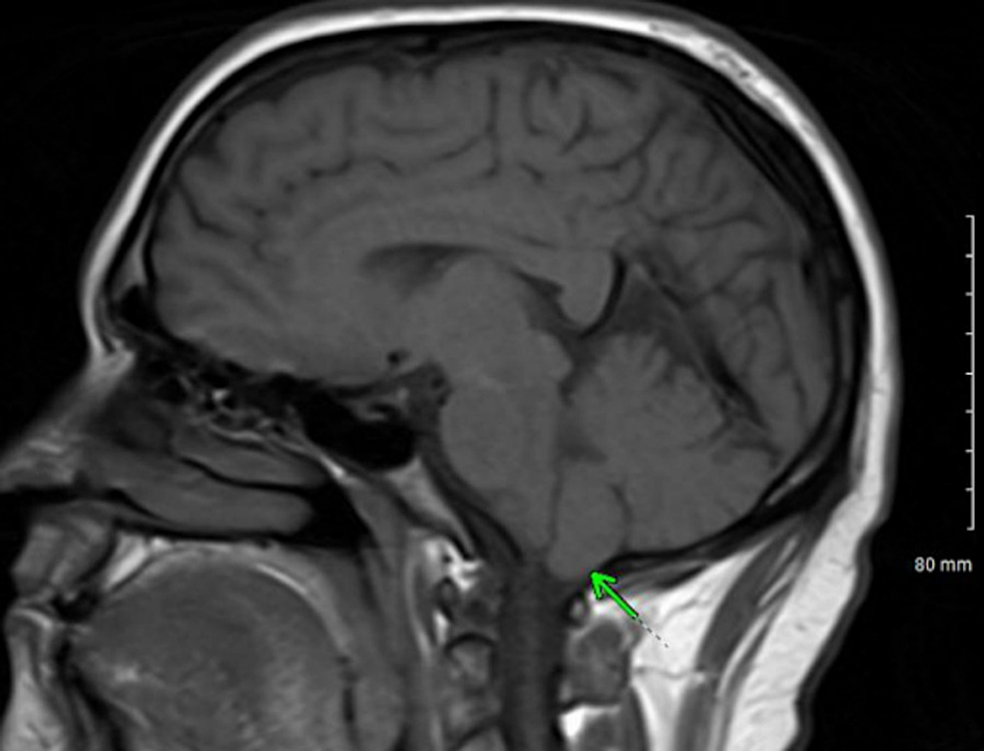
T1 (sagittal view) showing low-lying cerebellar tonsils (green arrow)

Our patient had elevated CSF pressure of 260 mmH2O with MRI brain showing pathognomonic signs of elevated intracranial pressure, i.e., enlarged bilateral optic nerve sheaths (Figure 1), empty sella turcica (Figure 2), and low-lying cerebellar tonsils (Figure 3) [5].

MRV visualized stenosed left transverse and sigmoid sinus [5]. Anti-MOG (myelin oligodendrocyte glycoprotein) and NMO (Neuromyelitis optica) antibodies were negative. There are no demyelinating lesions or abnormal enhancements in the MRI brain, cervical, and thoracic spine. All mentioned findings ruled out secondary causes of raised intracranial pressure, including tumors, strokes, infections, multiple sclerosis (MS), myelin oligodendrocyte glycoprotein antibody disorders (MOGAD), and neuromyelitis Optica (NMO).

Although PTCS' pathophysiology is a topic of debate, different mechanisms involving the abnormal dynamics of CSF flow have been postulated over the last century. More relevant include increased CSF production, impaired CSF absorption, and elevated cerebral venous pressure [6-8].

With eight restricted OCBs in CSF (two separate nonrestricted OCBs in serum and CSF), myelin essential protein (MBP) elevated at 4.4 ng/mL, immunoglobulin G (IgG) synthesis rate of 53.7 mg/day, and IgG index 2.9; we also question the role of immune-mediated pathogenesis of PTCS. An extensive prospective series investigating OCBs in PTCS is by Altıokka-Uzun G et al. [9]. They reported frequency of vision loss was significantly higher in the group of PTCS with positive OCBs compared to negative OCBs [9]. They also found patients with PTCS had highly elevated tumor necrosis factor-α (TNF-α), interferon- γ (IFN-γ), and interleukins 4,10,12,17 in their serum compared to patients with multiple sclerosis (MS), further strengthening the evidence of an immunological role in PTCS [9].

Another query yet to be answered is the role of obesity and inflammatory markers in PTCS. Arguably, obesity is an inflammatory state. One theory hypothesizes that fat tissues synthesize pro-inflammatory markers and OCBs, which pass from serum to CSF as seen in the rare disease "rapid-onset obesity with hypothalamic dysfunction hypoventilation, and autonomic dysregulation syndrome" [9-10]. However, Altıokka-Uzun G et al. also found TNF-α and IL-17, higher in CSF than serum concentrations in PTCS, indicating the intrathecal presence of cytokine-producing immune cells and further quizzing the role of B and T cells inside CSF. 

Research by El-Tamawy, M.S. et al. studying immunological markers and OCBs in Egyptian patients showed the duration of illness being longer in OCB positive patients with a mean of (27.5 + 15.92 months) than in OCB negative patients [11]. In addition, the TNF-α level was also significantly higher in OCB positive group, correlating with the Altıokka-Uzun G et al. finding.

Despite some evidence of immunological markers in PTCS, first-line medical treatment remains acetazolamide, along with weight loss [2,10]. On the other hand, topiramate has shown promising results, as it also causes significant weight loss and is studied to be as effective as acetazolamide [10]. In addition, high-dose pulsatile steroids have been suggested as a temporary option in patients with acute worsening of vision till they get surgically fixed [12]. Surgical treatment for medically refractory and worsening visual loss is A) optic nerve sheath fenestration (ONFS) and B) CSF diversion via ventriculoperitoneal shunt (VP shunt) or lumboperitoneal shunt (LS). However, the first line of surgical therapy amongst these is variable [13]. 

The patient was refractory to high-dose acetazolamide. He went through right eye ONFS with improvement in peripheral vision, and after one month, ONFS in the left eye with no progress so far.

Our case is unique, as the patient had a gradual bilateral visual loss with papilledema and without headaches, tinnitus, hemifacial spasms, weakness, or cranial nerve palsies, especially, no abducens nerve palsy. At the same time, his CSF workup was novel for eight restricted oligoclonal bands (OCBs) in the CSF and two separate non-restricted OCBs in serum and CSF; the highest number reported of restricted OCBs in PTCS without a secondary cause as per our knowledge. Data regarding similar case reports on PTCS without secondary cause and OCBs are scarce. A similar case to ours was reported recently of a 33-year-old, morbidly obese woman who developed progressively worsening blurry vision with papilledema and temporal headache. CSF had 15 OCBs, and with the help of radiological findings, she was diagnosed with an overlap of MS and IIH [14].

## Conclusions

Pseudotumor cerebri syndrome's exact pathophysiology is unknown. However, evolving data regarding multiple inflammatory markers found in CSF also suggest immunological involvement in PTCS pathogenesis. Males with PTCS can present without headache and tinnitus and overall have a higher propensity toward visual loss, making early ophthalmological exams crucial for a better prognosis. Future trials of OCB-positive PTCS patients may help prove an association of OCB with the duration and severity of the disease.
